# Splenic abscesses complicating acute septicemic melioidosis

**DOI:** 10.1590/0037-8682-0394-2023

**Published:** 2023-09-22

**Authors:** Chee Yik Chang

**Affiliations:** 1 Hospital Sungai Buloh, Medical Department, Selangor, Malaysia.

**FIGURE 1:**
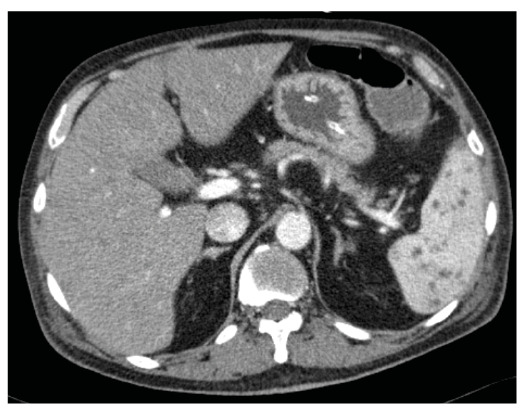
Computed tomography scan of the thorax and abdomen showing the presence of multiple splenic microabscesses.

A 60-year-old male farmer with newly diagnosed type 2 diabetes mellitus presented with a 3-day history of high-grade fever and progressive dyspnea. Upon arrival at our hospital, he experienced septicemic shock and severe respiratory failure, which led to emergency intubation. Urgent computed tomography (CT) of the thorax and abdomen revealed bilateral lung consolidation and multiple splenic microabscesses (Figure 1). *Burkholderia pseudomallei* was isolated from multiple blood cultures and was found to be susceptible to amoxicillin-clavulanic acid, ceftazidime, imipenem, and trimethoprim-sulfamethoxazole. The patient was diagnosed with disseminated septicemic melioidosis, pneumonia, and a splenic abscess. The patient was administered intravenous meropenem (1 g every 8 h) and subsequently improved clinically. 

Melioidosis, also known as Whitmore disease, is a potentially fatal disease that is difficult to diagnose because its clinical presentation mimics that of many other diseases. It can present with diverse clinical manifestations, including pneumonia, genitourinary infections, visceral abscesses, skin and soft tissue infections, septic arthritis, neurological melioidosis, and fulminant septicemia, without a clear focus. Melioidosis is frequently associated with internal organ abscess. In the Darwin prospective study, splenic abscesses (5%) were the second most common type of internal organ abscesses after prostatic abscesses (20%)^1^. *Burkholderia pseudomallei* is the most common causative agent of splenic abscesses in melioidosis-endemic areas^2^. Bedside ultrasonography has been advocated for the detection of internal organ abscesses in febrile patients in endemic areas, enabling the prompt initiation of empirical antibiotic therapy for melioidosis^2^ ^,^ ^3^.
